# Cardiovascular Events in Individuals Treated With Sulfonylureas or Dipeptidyl Peptidase 4 Inhibitors

**DOI:** 10.1001/jamanetworkopen.2025.23067

**Published:** 2025-07-24

**Authors:** Alexander Turchin, Lucia C. Petito, Emma Hegermiller, Ryan Carnahan, Andrea DeVries, Satyender Goel, M. Cecilia Lansang, Marie E. McDonnell, Vinit Nair, Elisa Priest, Vincent J. Willey, Alan F. Kaul, Miguel A. Hernán

**Affiliations:** 1 Division of Endocrinology, Brigham and Women’s Hospital, Boston, Massachusetts; 2 Harvard Medical School, Boston, Massachusetts; 3 Northwestern University Feinberg School of Medicine, Chicago, Illinois; 4 Indus Consulting, Inc, Chicago, Illinois; 5 The University of Iowa College of Public Health, Iowa City; 6 Humana Healthcare Research, Inc, Louisville, Kentucky; 7 Cleveland Clinic, Cleveland, Ohio; 8 Baylor Scott and White Research Institute, Dallas, Texas; 9 Carelon Research, Wilmington, Delaware; 10 Medical Outcomes Management, Inc, Sharon, Massachusetts; 11 CAUSALab, Harvard T.H. Chan School of Public Health, Boston, Massachusetts

## Abstract

**Importance:**

Sulfonylureas are commonly used to treat type 2 diabetes (T2D). Research findings on cardiovascular risk associated with sulfonylureas have been inconsistent.

**Objective:**

To emulate a target trial that compares the risk of cardiovascular events after initiation of treatment with individual sulfonylureas or dipeptidyl peptidase 4 inhibitors (DPP4is).

**Design, Setting, and Participants:**

This comparative effectiveness research study included individuals with T2D and moderate cardiovascular risk treated with metformin monotherapy who received care at 1 of 10 US health systems or were insured by 1 of 2 large health insurance plans between January 1, 2014, and January 1, 2023. Data were analyzed from July 2024 to March 2025.

**Exposure:**

Initiation of treatment with a sulfonylurea (glimepiride, glipizide, or glyburide) or a DPP4i (reference category) as a second line therapy after metformin.

**Main Outcomes and Measurements:**

The primary outcome was a 4-point composite of major adverse cardiovascular events (MACE-4): myocardial infarction, ischemic stroke, heart failure hospitalization, or cardiovascular death (from any of these conditions). The 5-year risks of each outcome were estimated.

**Results:**

Among 48 165 eligible individuals (median [IQR] age, 61 [52-69] years; 22 674 female [47.1%]; median [IQR] hemoglobin A_1C_, 7.8% [7.3%-8.5%]; median [IQR] low-density lipoprotein cholesterol, 89 mg/dL [70-112 mg/dL]), 18 147 started glipizide, 14 282 started glimepiride, 1887 started glyburide, and 13 849 started a DPP4i. Over the median (IQR) follow-up of 37 (20-64) months, 3158 individuals (6.6%) experienced a MACE-4. The estimated 5-year risks of MACE-4 were 8.1% (95% CI, 7.5%-8.7%) for DPP4i, 8.4% (95% CI, 6.8%-9.9%) for glyburide, 8.6% (95% CI, 7.9%-9.2%) for glimepiride, and 9.1% (95% CI, 8.7%-9.7%) for glipizide. Compared with DPP4is, the 5-year risk ratio of MACE-4 was 1.13 (95% CI, 1.03-1.23) for glipizide, 1.07 (95% CI, 0.96-1.16) for glimepiride, and 1.04 (95% CI, 0.83-1.24) for glyburide.

**Conclusions and Relevance:**

In this comparative effectiveness research study of sulfonylureas vs DPP4i in patients with T2D, the risk of MACE-4 events was highest for glipizide. These findings suggest that sulfonylureas, glipizide in particular, may not be the optimal agent in treatment of individuals with T2D at moderate cardiovascular risk.

## Key Points

**Question:**

Do any sulfonylureas (glimepiride, glipizide, or glyburide) associated increase the risk of cardiovascular events compared with dipeptidyl peptidase 4 inhibitors (DPP4is)?

**Findings:**

In this comparative effectiveness research study of electronic health records and claims from 48 165 individuals starting a second T2D agent after metformin, individuals treated with glipizide had a higher risk of a composite end point of myocardial infarction, ischemic stroke, heart failure hospitalization, and cardiovascular death than those treated with a DPP4i.

**Meaning:**

These findings suggest that sulfonylureas, glipizide in particular, may not be the optimal agents in treatment of individuals with T2D at moderate cardiovascular risk.

## Teaser

This comparative effectiveness research study compares the risk of cardiovascular events after initiation of treatment with individual sulfonylureas or dipeptidyl peptidase 4 inhibitors (DPP4is).

## Introduction

Type 2 diabetes (T2D) is a common chronic disease whose prevalence continues to grow in the US and worldwide.^1,2^ Individuals with T2D have an increased risk of adverse cardiovascular events, including coronary ischemia, stroke, and heart failure (HF).^3,4,5,6,7,8^ Mitigation of cardiovascular risk is therefore an important aspect of treatment of T2D.^9^

Sulfonylureas remain a common choice of pharmacotherapy of T2D,^10,11^ due in part to their low cost and glucose-lowering potency.^12^ In the past, individual sulfonylureas that are no longer available in the US (eg, tolbutamide) were found to increase the risk of cardiovascular events.^13^ It is not known whether treatment with any sulfonylureas currently in clinical use increases cardiovascular risk compared with other therapeutic alternatives.

A long-term randomized clinical trial to compare individual sulfonylureas currently in use in the US would be resource and time-intensive and may never be conducted. We therefore leveraged a multicenter observational study of individuals with T2D to emulate a target trial that compares the incidence of cardiovascular events after initiation of treatment with individual sulfonylureas and dipeptidyl peptidase 4 inhibitors (DPP4is) as a second line agent after metformin.

## Methods

### Target Trial Specification

This comparative effectiveness research study followed the Strengthening the Reporting of Observational Studies in Epidemiology (STROBE) reporting guideline and was approved by the institutional review boards of all participating institutions. The requirement of informed consent was waived due to the use of deidentified data. The elements of the protocol of the target trial are summarized in eTable 1 in Supplement 1. Individuals are eligible for a target trial if they meet the following criteria between January 1, 2014, and January 1, 2023: (1) aged 30 years or older; (2) diagnosis of T2D and no evidence of diabetes of any other type; (3) hemoglobin A_1C_ (HbA_1C_) between 7.0% and 11% (to convert to proportion of total hemoglobin, multiply by 0.01); (4) monotherapy with metformin for at least 3 months and no prior nonmetformin outpatient diabetes therapy; (5) moderate cardiovascular risk; (5) no metastatic cancer, end-stage lung disease, end-stage liver disease, dementia, previous history of pancreatitis, previous history of medullary thyroid cancer, previous history of pyelonephritis, or estimated glomerular filtration rate (eGFR) less than 45 mL/min/1.73 m^2^; (6) at least 12 months of engagement with their health system or continuous enrollment at their health insurance plan to ensure availability of historical information on baseline characteristics and to increase the probability of continued engagement; and (7) a clinician’s determination that a second-line treatment needs to be initiated. Moderate cardiovascular risk is defined as: (1) no history of diagnosis of atherosclerotic cardiovascular disease or hospitalization for HF and either (2) males aged 35 years and older and females aged 45 years or older or (3) males aged 30 to 34 years and females aged 30 to 44 years with hypertension, hyperlipidemia, retinopathy, kidney disease, or neuropathy.^14^

The treatment strategies of interest include immediate initiation of exactly 1 of the following medications after metformin: (1) glimepiride, (2) glipizide, (3) glyburide, or (4) any DPP4i (alogliptin, linagliptin, saxagliptin, or sitagliptin). Individuals can subsequently continue or discontinue study medications at their discretion. DPP4is are chosen as the reference category because multiple randomized clinical trials have shown no difference in all or most cardiovascular events between each of the DPP4is and placebo.^15,16,17,18,19,20^ In a variation of this target trial, saxagliptin was excluded from the DPP4i category because a previous randomized trial showed an increased risk of HF in individuals treated with saxagliptin.^19^

The outcome is the 4-point composite of major adverse cardiovascular events (MACE-4): (1) myocardial infarction (MI), (2) ischemic stroke, (3) HF hospitalization, or (4) death from any of these conditions. Individual components of the composite end point are also outcomes of interest. Follow-up starts at assignment and ended at the outcome, loss to follow-up (12 months without health care encounters, vital signs, laboratory measurements, or loss of membership with the health insurance plan), or at study end (July 1, 2023). For MACE-4 and cardiovascular death, noncardiovascular mortality is a competing event; for MI, stroke, and HF, the competing event is non-MI, nonstroke, and non-HF mortality, respectively. The causal contrast of interest is the intention-to-treat effect, defined as the total effect.^21^

In the intention-to-treat analysis, the 5-year cumulative incidence (referred to as risk) of each outcome is compared between treatment strategies via ratios and differences.^22^ Risks for each outcome are estimated nonparametrically via a Kaplan-Meier estimator or parametrically via a pooled logistic regression model^23^ for the monthly probability of that outcome with the following covariates: month of follow-up (modeled as a quadratic polynomial), indicators for treatment strategy, and product (interaction) terms between linear month and the treatment indicators. Prespecified prognostic factors are also included as covariates in the model, with the risk estimates standardized to the distribution of these factors. Subgroup analyses for the MACE-4 outcome are conducted by sex, age, baseline HbA_1C_, and baseline eGFR. Nonparametric bootstrap with 500 resamples are used to calculate 95% CIs for all estimates.

### Target Trial Emulation

We emulated the target trial using data from the Observational Evaluation of Second Line Therapy Medications in Diabetes (BESTMED) study, which includes individuals with T2D who were treated at 1 of 10 health systems or had their health insurance at 1 of 2 national health insurance plans in the US. Data were harmonized across study sites using the PCORnet Common Data Model.^24^ Individuals who had data at more than 1 study site were identified using a privacy-preserving linkage solution^25^ and their records were merged for the analysis.

The target trial focused on individuals at moderate cardiovascular risk in part to reduce the confounding that could result if any of the study medications were less likely to be prescribed to individuals at high cardiovascular risk. Information on individuals’ baseline characteristics, analytical covariates, and outcomes was obtained from electronic health records of participating health systems and claims data of participating health insurance plans (combined with electronic health record and laboratory data of their data partners). Race and ethnicity information was collected via via patient registration, available through the electronic health record and health insurance plan data and was included to account for a known disparity. Race categories included Asian, Black, White, and other (ie, persons having origins in any of the original peoples of North and South America [including Central America] and who maintain tribal affiliation or community attachment, persons having origins in any of the original peoples of Hawaii, Guam, Samoa, or other Pacific Islands, and persons categorized with multiple races or any race not otherwise specified in their medical records). Ethnicity categories included Hispanic and non-Hispanic. Information on medications was obtained from prescription records (health care delivery organizations) and prescription fills (health insurance plans). Treatment adherence was assessed using both prescription and dispensing records. Prescriptions were considered discontinued 1 year after the last prescription (based on the typical refill duration in the US). Dispensing treatment windows were calculated based on the date of drug dispensing and the recorded days’ supply. Information on the cause of death was obtained from the National Death Index for individuals with evidence of death in health insurance plan or electronic health record data or a nationwide mortality database (derived from public and commercial data sources using a previously validated methodology^26^).

### Statistical Analysis

The 5-year risks of each outcome were estimated as described for the target trial. The pooled logistic regression models included the following baseline covariates: demographics (age, sex, and health insurance); community factors (proportion treated with brand name vs generic anticoagulants at the individual’s site and Social Deprivation Index^27^ in the individual’s zip code); laboratory values (HbA_1C_, eGFR, low density lipoprotein cholesterol); excess weight (body mass index [BMI; calculated as weight in kilograms divided by height in meters squared] ≥35, or *International Statistical Classification of Diseases and Related Health Problems, Tenth Revision [ICD-10]* code indicating class II-III obesity); proxies of comorbidity load (number of nondiabetes medications the patient was taking, number of outpatient visits, and number of distinct laboratory tests in the previous year and hospitalization in the previous 3 months); history of HF; history of metabolic dysfunction–associated steatotic liver disease and metabolic dysfunction–associated steatohepatitis; Charlson comorbidity index; and treatment with systemic glucocorticoids, statins, or antiplatelet drugs. eTable 2 in Supplement 1 describes these variables and the methods used to address missing data.

We also conducted several sensitivity analyses. First, to explore the impact of confounding adjustment on our estimates, we obtained estimates adjusted for age only. Second, to explore the impact of the handling of competing events, we censored individuals when they experience the competing event (death from any cause). Third, to explore the sensitivity of our analysis to our method of addressing missing data, we added indicators for missingness (details in eTable 3 in Supplement 1). Fourth, to explore the impact of adjustment for hypertension, a prevalent co-occurring comorbidity, we added hypertension (identified by *ICD-10* codes and blood pressure, as available) as a confounder in the outcome model. Finally, to assess that the impact of including individuals with history of HF or atrial fibrillation, we conducted an analysis excluding individuals who had these conditions at baseline. All statistical analyses were performed in R version 4.4.0 from July 2024 to March 2025.

## Results

### Study Population

**Figure 1.**
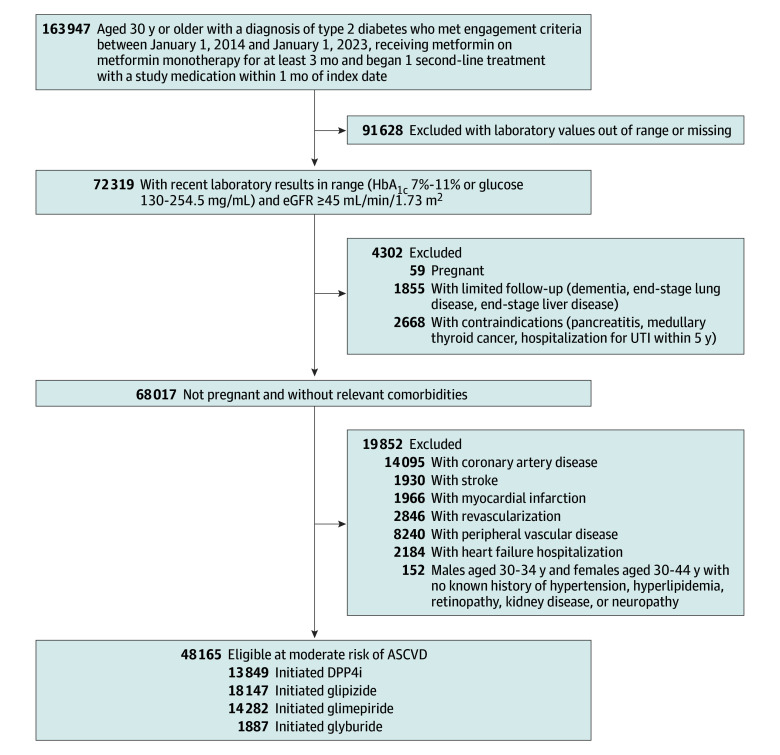
Selection of Individuals for the Target Trial Emulation, Observational Evaluation of Second Line Therapy Medications in Diabetes Study, 2014-2023 ASCVD indicates atherosclerotic cardiovascular disease; DPP4i, dipeptidyl peptidase-4 inhibitors; eGFR, estimated glomerular filtration rate; HbA_1C_, hemoglobin A_1C_; UTI, urinary tract infection. To convert HbA_1C_ to proportion of total hemoglobin, multiply by 0.01; to convert glucose to millimoles per liter, multiply by 0.0555.

Of 48 165 eligible individuals (median [IQR] age, 61 [52-69] years; 22 674 female [47.1%]; median [IQR] HbA_1C_, 7.8% [7.3%-8.5%]; median [IQR] low-density lipoprotein cholesterol, 89 mg/dL [70-112 mg/dL]), 18 147 initiated glipizide, 14 282 initiated glimepiride, 1887 initiated glyburide, and 13 849 initiated a DPP4i (Figure 1). Differences in the median values of baseline characteristics between individuals treated with any of the 3 sulfonylureas vs DPP4i (the reference category) did not exceed 2 years for age, 0.2% for HbA_1C_, 7 mg/dL (to convert to millimoles per liter, multiply by 0.0259) for low-density lipoprotein cholesterol, and 3 mL/min/1.73 m^2^ for eGFR (Table 1). The median (IQR) time these individuals continued treatment with the study drug was 13 (6-29) months for glipizide, 14 (6-32) months for glimepiride, 11 (3-20) months for glyburide, and 12 (5-23) months for DPP4is. See eTable 4 in Supplement 1 for baseline characteristics and study medication prescribing for eligible individuals by study site.

**Table 1.** Baseline Characteristics of Eligible Individuals in the Observational Evaluation of Second Line Therapy Medications in Diabetes Study, 2014-2023

Characteristic	Participants, No. (%)					SMD (vs DPP4i)		
	Overall (N = 48 165)	DPP4i (n = 13 849)	Glimepiride (n = 14 282)	Glipizide (n = 18 147)	Glyburide (n = 1887)	Glimepiride	Glipizide	Glyburide
Age, median (IQR), y	61 (52-69)	60 (52- 68)	62 (53-69)	61 (52-69)	58 (50-66)	0.13	0.07	0.18
Sex								
Female	22 674 (47.1)	6813 (49.2)	6484 (45.4)	8444 (46.5)	933 (49.4)	0.08	0.05	0.00
Male	25 491 (52.9)	7036 (50.8)	7798 (54.6)	9703 (53.5)	954 (50.6)			
Race								
Asian	1108 (2.3)	300 (2.2)	305 (2.1)	469 (2.6)	34 (1.8)	0.37	0.34	0.22
Black	3824 (7.9)	1053 (7.6)	1032 (7.2)	1580 (8.7)	159 (8.4)			
White	26 132 (54.3)	6088 (44.0)	8613 (60.3)	10 433 (57.5)	998 (52.9)			
Other^a^	2107 (4.4)	535 (3.9)	613 (4.3)	868 (4.8)	91 (4.8)			
No information	14 994 (31.1)	5873 (42.4)	3719 (26.0)	4797 (26.4)	605 (32.1)			
Ethnicity								
Hispanic	2623 (5.4)	650 (4.7)	757 (5.3)	1059 (5.8)	157 (8.3)	0.37	0.35	0.24
Non-Hispanic	30 407 (63.1)	7221 (52.1)	9812 (68.7)	12 263 (67.6)	1111 (58.9)			
No information	15 135 (31.4)	5978 (43.2)	3713 (26.0)	4825 (26.6)	619 (32.8)			
Private health insurance	38 487 (79.9)	11 738 (84.8)	11 025 (77.2)	14 312 (78.9)	1412 (74.8)	0.19	0.15	0.25
SDI percentile								
1-30	13 821 (32.2)	3761 (32.1)	4015 (31.0)	5557 (33.4)	488 (29.4)	0.03	0.05	0.07
31-60	12 736 (29.6)	3376 (28.8)	3913 (30.3)	4979 (29.9)	468 (28.2)			
61-100	16 409 (38.2)	4582 (39.1)	5004 (38.7)	6120 (36.7)	703 (42.4)			
Hospitalization in previous 3 mo	1256 (2.6)	389 (2.8)	361 (2.5)	457 (2.5)	49 (2.6)	0.02	0.02	0.01
HbA_1C_, median (IQR), %	7.8 (7.3-8.5)	7.7 (7.3-8.4)	7.8 (7.4-8.5)	7.8 (7.4-8.6)	7.9 (7.4-8.7)	0.06	0.11	0.21
eGFR, median (IQR), mL/min/1.73 m^2^	91.0 (76.5-101.8)	91.6 (77.2-102.3)	90.5 (76.2-101.1)	90.5 (76.0-101.4)	94.4 (79.1-104.9)	0.05	0.06	0.13
LDL cholesterol, median (IQR), mg/dL	89 (70-112)	88 (69-111)	89 (70-112)	90 (70-113)	95 (76-118)	0.01	0.04	0.20
BMI, median (IQR)^b^	34.0 (29.8-39.0)	33.1 (29.0-38.6)	34.0 (30.0-39.0)	34.0 (30.0-39.0)	33.8 (30.0-39.0)	0.06	0.09	0.05
CCI, mean (SD)	2.13 (1.77)	2.11 (1.76)	2.12 (1.77)	2.16 (1.79)	1.99 (1.72)	0.00	0.03	0.07
CCI ≥5	1685 (3.5)	507 (3.7)	487 (3.4)	637 (3.5)	54 (2.9)	0.01	0.01	0.04
Smoking status								
Nonsmoker	8424 (17.5)	1944 (14.0)	2575 (18.0)	3532 (19.5)	373 (19.8)	0.29	0.28	0.26
Current smoker	9068 (18.8)	1808 (13.1)	3162 (22.1)	3734 (20.6)	364 (19.3)			
No information	30 673 (63.7)	10 097 (72.9)	8545 (59.8)	10 881 (60.0)	1150 (60.9)			
Hypertension	38 516 (80.0)	11 201 (80.9)	11 456 (80.2)	14 419 (79.5)	1440 (76.3)	0.02	0.04	0.11
Heart failure	2828 (5.9)	754 (5.4)	851 (6.0)	1111 (6.1)	112 (5.9)	0.02	0.03	0.02
Atrial fibrillation	1471 (3.1)	415 (3.0)	453 (3.2)	560 (3.1)	43 (2.3)	0.01	0.00	0.04
Hypoglycemia in the previous 5 y	1488 (3.1)	444 (3.2)	417 (2.9)	570 (3.1)	57 (3.0)	0.02	0.00	0.01
Statin therapy	33 362 (69.3)	9572 (69.1)	9879 (69.2)	12 748 (70.2)	1163 (61.6)	0.00	0.03	0.16
Antiplatelet therapy	4564 (9.5)	1125 (8.1)	1306 (9.1)	1997 (11.0)	136 (7.2)	0.04	0.10	0.03

Abbreviations: BMI, body mass index; CCI, Charlson Comorbidity Index; DPP4i, dipeptidyl peptidase-4 inhibitor; eGFR, estimated glomerular filtration rate; HbA_1C_, hemoglobin A_1C_; LDL, low-density lipoprotein; SDI, Social Deprivation Index; SMD, standardized mean difference.

SI conversion factor: To convert HbA_1C_ to proportion of total hemoglobin, multiply by 0.01; LDL cholesterol to millimoles per liter, multiply by 0.0259.

^a^ Persons having origins in any of the original peoples of North and South America (including Central America) and who maintain tribal affiliation or community attachment, persons having origins in any of the original peoples of Hawaii, Guam, Samoa, or other Pacific Islands, and persons categorized with multiple races or any race not otherwise specified in their medical records.

^b^ Calculated as weight in kilograms divided by height in meters squared.

### Cardiovascular Events

Over a median (IQR) follow-up of 37 (20-64) months, 3158 (6.6%) individuals had a MACE-4, 1082 (2.2%) had an MI, 1260 (2.6%) had an ischemic stroke, 1460 (3.0%) had an HF hospitalization, and 206 (0.4%) died from any of these conditions, while 1475 individuals (3.1%) died from noncardiovascular causes and 17 168 individuals (35.6%) were lost to follow-up. See eTable 5 in Supplement 1 for number of events by treatment group and cardiovascular outcome.

**Figure 2.**
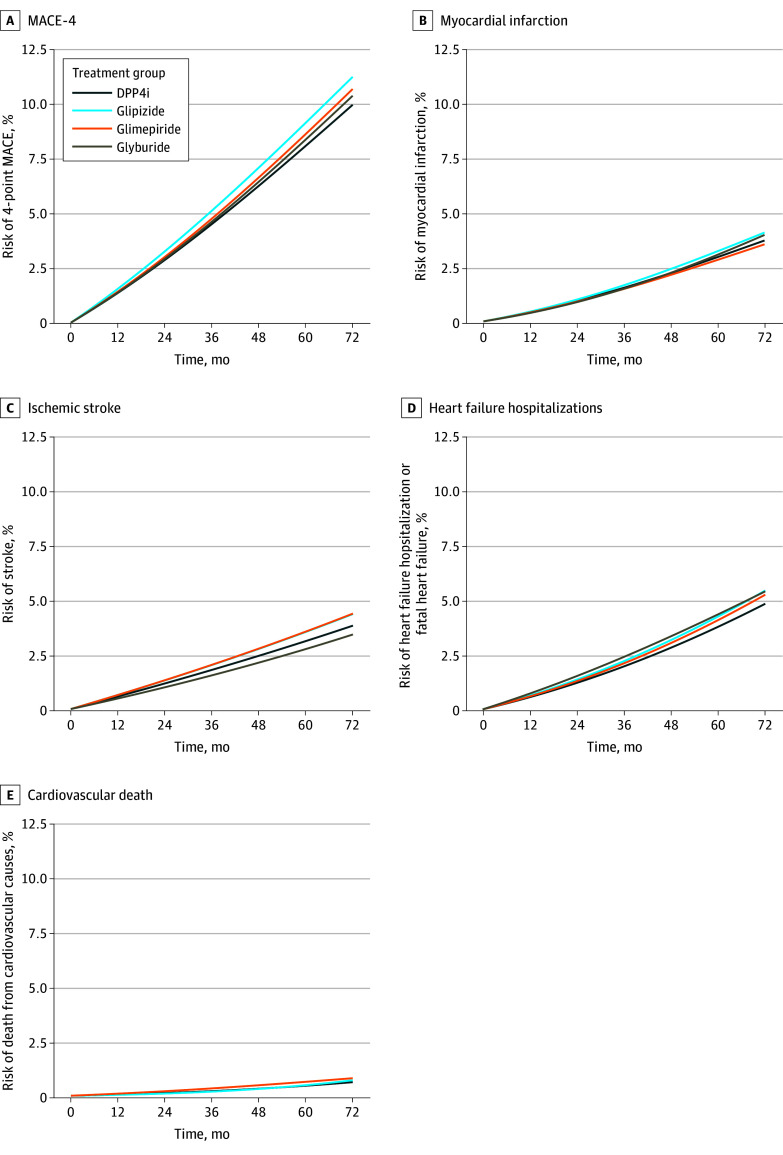
Cumulative Incidence of Cardiovascular Outcomes by Treatment Group Both fatal and nonfatal events are included in panels B, C, and D. In panel C, the blue line (glipizide) cannot be easily seen because it almost fully overlaps with the orange line (glimepiride). DPP4i indicates dipeptidyl peptidase-4 inhibitor; MACE-4, major adverse cardiovascular events (4-point composite).

**Figure 3.**
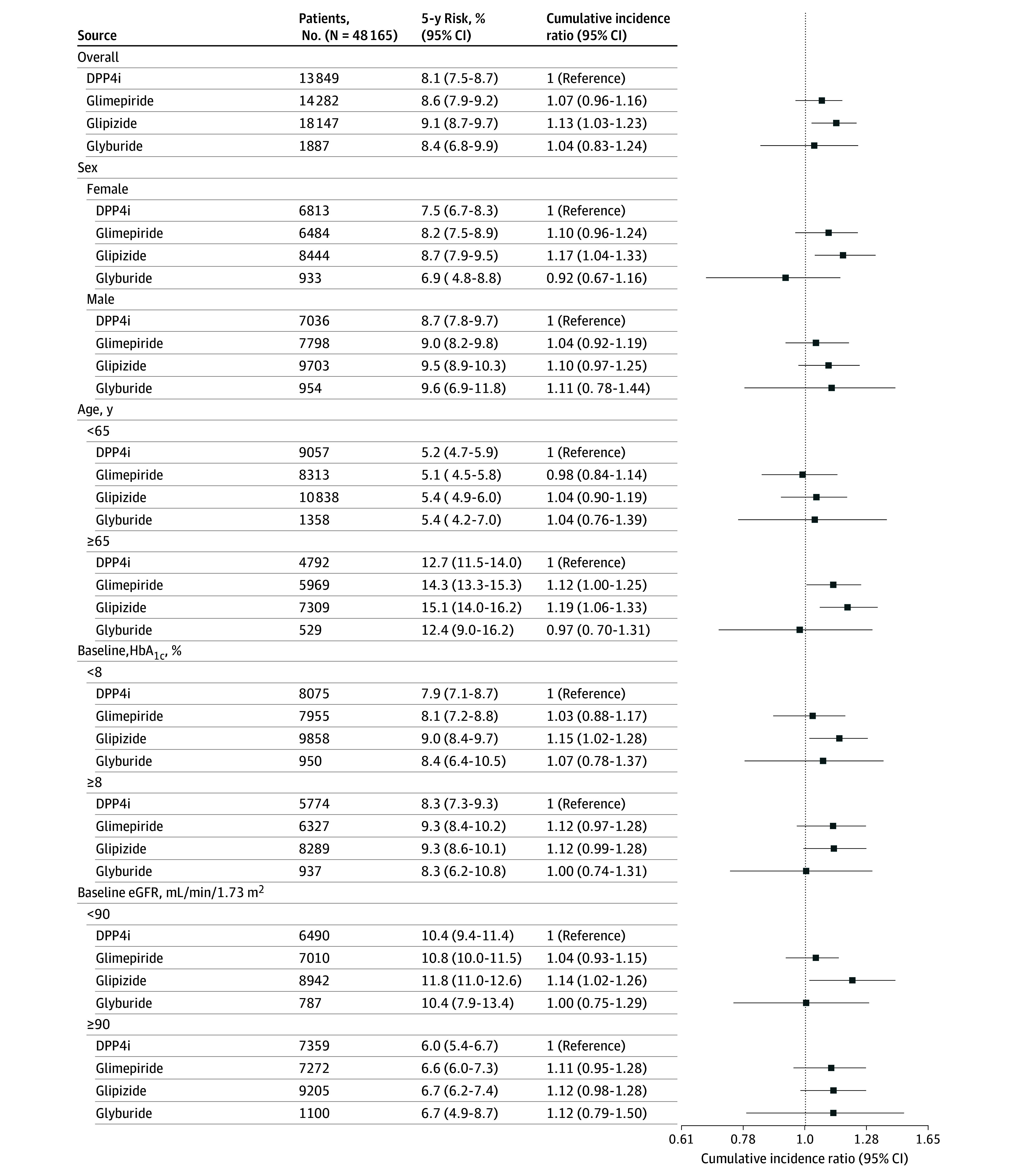
Subgroup Analyses for the Primary Outcome DPP4i indicates dipeptidyl peptidase-4 inhibitor; eGFR, estimated glomerular filtration rate; HbA_1C_, hemoglobin A_1C_. To convert HbA_1C_ to proportion of total hemoglobin, multiply by 0.01.

The estimated 5-year risks of MACE-4 were 8.1% (7.5%-8.7%) for DPP4is, 8.4% (6.8%-9.9%) for glyburide, 8.6% (7.9%-9.2%) for glimepiride, and 9.1% (8.7%-9.7%) for glipizide. Compared with DPP4is, the 5-year risk ratios of MACE-4 were 1.04 (95% CI, 0.83-1.24) glyburide, 1.07 (95% CI, 0.96-1.16) for glimepiride, and 1.13 (95% CI, 1.03-1.23) for glipizide (Figure 2 and Table 2). Point estimates were similar across subgroups defined by sex, age, HbA_1C_, and eGFR, although confidence intervals were wide (Figure 3).

**Table 2.** Estimated 5-Year Risks of Cardiovascular Outcomes by Treatment Group, Observational Evaluation of Second Line Therapy Medications in Diabetes Study 2014-2023^a^

Outcome by treatment group	No. eligible	No. of events at 5-y	5-y Risk, % (95% CI)	Risk difference, % (95% CI)	Risk ratio (95% CI)	Average hazard ratio (95% CI)
4-point MACE						
DPP4i	13 849	663	8.1 (7.5 to 8.7)	0 [Reference]	1 [Reference]	1 [Reference]
Glimepiride	14 282	824	8.6 (7.9 to 9.2)	0.54 (−0.35 to 1.25)	1.07 (0.96 to 1.16)	1.05 (0.92 to 1.17)
Glipizide	18 147	1021	9.1 (8.7 to 9.7)	1.06 (0.23 to 1.80)	1.13 (1.03 to 1.23)	1.14 (1.01 to 1.27)
Glyburide	1887	83	8.4 (6.8 to 9.9)	0.29 (−1.37 to 1.84)	1.04 (0.83 to 1.24)	1.02 (0.77 to 1.29)
Stroke^b^						
DPP4i	13 879	266	3.1 (2.8 to 3.5)	0 [Reference]	1 [Reference]	1 [Reference]
Glimepiride	14 312	349	3.5 (3.2 to 3.9)	0.43 (−0.02 to 0.88)	1.14 (0.99 to 1.31)	1.13 (0.94 to 1.38)
Glipizide	18 192	388	3.5 (3.2 to 3.8)	0.42 (−0.05 to 0.88)	1.14 (0.98 to 1.31)	1.13 (0.96 to 1.36)
Glyburide	1893	27	2.7 (1.8 to 4.0)	−0.36 (−1.39 to 0.9)	0.88 (0.56 to 1.29)	0.86 (0.49 to 1.30)
Heart failure hospitalization or fatal heart failure						
DPP4i	13 881	290	3.7 (3.3 to 4.1)	0 [Reference]	1 [Reference]	1 [Reference]
Glimepiride	14 317	372	4 (3.7 to 4.4)	0.3 (−0.21 to 0.82)	1.08 (0.95 to 1.24)	1.07 (0.90 to 1.28)
Glipizide	18 198	458	4.2 (3.8 to 4.6)	0.47 (−0.04 to 0.96)	1.12 (0.99 to 1.26)	1.12 (0.94 to 1.30)
Glyburide	1892	43	4.3 (3.3 to 5.6)	0.56 (−0.57 to 1.87)	1.15 (0.85 to 1.53)	1.24 (0.84 to 1.77)
Myocardial infarction^b^						
DPP4i	13 880	231	3.0 (2.6 to 3.3)	0 [Reference]	1 [Reference]	1 [Reference]
Glimepiride	14 316	256	2.8 (2.5 to 3.2)	−0.13 (−0.72 to 0.30)	0.96 (0.78 to 1.11)	0.97 (0.75 to 1.16)
Glipizide	18 203	348	3.2 (2.9 to 3.6)	0.27 (−0.26 to 0.80)	1.09 (0.92 to 1.29)	1.07 (0.89 to 1.31)
Glyburide	1896	31	3.1 (2.2 to 4.2)	0.11 (−0.93 to 1.10)	1.04 (0.71 to 1.34)	0.96 (0.62 to 1.44)
Death from cardiovascular causes						
DPP4i	13 898	34	0.5 (0.3 to 0.6)	0 [Reference]	1 [Reference]	1 [Reference]
Glimepiride	14 332	64	0.6 (0.5 to 0.8)	0.18 (−0.05 to 0.39)	1.39 (0.92 to 2.19)	1.67 (1.03 to 2.91)
Glipizide	18 226	50	0.5 (0.3 to 0.6)	0.02 (−0.17 to 0.21)	1.05 (0.74 to 1.74)	0.88 (0.54 to 1.64)
Glyburide^c^	1897	4	0.7 (0.3 to 1.1)	0.28 (−0.11 to 0.71)	1.71 (0.73 to 3.16)	1.71 (0.73 to 3.18)

Abbreviations: DPP4i, dipeptidyl peptidase-4 inhibitor; MACE, major adverse cardiovascular events.

^a^ Adjusted analysis for cardiovascular death could not be carried out for glyburide due to the small number of events.

^b^ Both fatal and nonfatal events are included.

^c^ Due to limited sample size, estimates are partially adjusted for age only.

The estimated 5-year risk of components of the MACE-4 outcome ranged from 0.5% (95% CI, 0.3%-0.6%) for cardiovascular death for individuals treated with DPP4is or glipizide to 4.3% (95% CI, 3.3%-5.6%) for HF hospitalization for individuals treated with glyburide (Table 2). The estimated 5-year RRs for these outcomes (Figure 2) were broadly consistent with those for MACE-4. See eFigure 1 in Supplement 1 for unadjusted survival curves for MACE-4 by treatment group and eFigure 2 in Supplement 1 for subgroup analyses for risk differences.

### Sensitivity Analyses

When 367 individuals taking saxagliptin were excluded from the DPP4i category, 5-year risk ratios of the primary outcome (compared with DPP4is) were 1.01 (95% CI, 0.79-1.30) for glyburide, 1.05 (95% CI, 0.92-1.18) for glimepiride, and 1.13 (95% CI, 1.01-1.26) for glipizide. The results of the analyses by individual end points were broadly similar (eTable 6 in Supplement 1). In particular, 5-year risk ratios for HF hospitalization (compared with DPP4is) were (1.07; 95% CI, 0.94-1.23) for glimepiride, 1.11 (95% CI, 0.98-1.30) for glipizide, and 1.13 (95% CI, 0.81-1.58) for glyburide. The results of other sensitivity analyses were also similar to those from the main analysis (eTable 7 in Supplement 1).

## Discussion

In this nationwide comparative effectiveness research study of individuals with T2D at moderate cardiovascular risk, we estimated that the risk of cardiovascular events increased after initiation of glipizide compared with DPP4is. Glipizide—currently the most commonly used sulfonylurea in the US^11^—was estimated to increase the risk of MACE-4 by approximately 13%, with risk increases of 13 ± 10% highly compatible with the data under standard conventional criteria. The risk ratio for glimepiride was 1.07, but the estimates for MACE-4 and its components were imprecise. The estimates for glyburide were closer to the null and more imprecise.

A strength of our study is the comparison of individual sulfonylureas with DPP4is, which are a clinical alternative to sulfonylureas that have been demonstrated to be neutral for cardiovascular events compared with placebo. Only one previous high-quality study—the Cardiovascular Outcome Study of Linagliptin vs Glimepiride in Type 2 Diabetes clinical trial—compared a sulfonylurea (glimepiride) to a DPP4i (linagliptin).^28^ This trial, involving approximately 6000 participants, estimated a hazard ratio for cardiovascular events (not including HF) of 0.98 (95% CI, 0.84-1.14)—a finding compatible with our results.^28^ Other investigations did not compare sulfonylureas with DPP4is; a randomized trial^29^ found a hazard ratio of 1.31 (95% CI, 1.05-1.69) for glipizide compared with metformin and a meta-analysis^30^ of 3 randomized trials estimated a relative risk of 0.84 (95% CI, 0.56-1.26) for glyburide compared with other sulfonylureas and insulin. Results from a meta-analysis^31^ that found mortality differences between sulfonylureas are hard to interpret because of the inclusion of observational studies with questionable methodology (eg, comparing prevalent users).

A 13% relative increase in cardiovascular risk, like the one estimated in this study, is potentially clinically impactful. Decreases in cardiovascular risk of this magnitude have generally been thought to be sufficient for approval of medications for prevention of cardiovascular events, particularly in high-risk populations.^32,33^ The estimated absolute increase of 1.1% in risk of cardiovascular events was modest in this population at moderate risk but could be greater in a high-risk population. This study’s findings therefore suggest caution in prescribing glipizide to individuals at moderate cardiovascular risk or established atherosclerotic cardiovascular disease.

Treatment with sulfonylureas also has other risks, in particular hypoglycemia. The risk of hypoglycemia is thought to be higher for glyburide than for shorter acting sulfonylureas.^30,34^ Choice between individual sulfonylureas should therefore be made in an individualized manner, balancing the risks and benefits based on the particular patient’s health profile.

The potentially different cardiovascular risk between individual sulfonylureas in our study underscores the importance of evaluating each agent in a particular pharmacological class on its own merits. In multiple instances, individual pharmaceuticals were found to have a different risk-benefit profile than other medications in the same class.^35,36,37^ Among sulfonylureas, in particular, tolbutamide was found to have a greater risk of cardiovascular events, and glyburide was found to have a higher risk of hypoglycemia.^13,30^ These findings caution against extrapolating results of studies evaluating a single agent to the entire class, and temper concerns about the use of research and development resources for the development of multiple agents in the same pharmacological class.^38^

In addition to pancreatic β cells, sulfonylurea receptors (SURs) are also found on cardiomyocytes (SUR 2A) and vascular smooth muscle cells (SUR 2B). Decrease in cardiac ischemic preconditioning after exposure to sulfonylureas has been found in animal experiments.^39^ This effect has been proposed as a possible mechanism for an increased cardiovascular risk from sulfonylureas.^40^ However, glipizide is not thought to have a greater affinity for either SUR 2A or SUR 2B vs pancreatic SURs (SUR 1) compared with other sulfonylureas.^41^ Furthermore, this mechanism would not explain possible differential effects on stroke. Further research is therefore needed to establish pathophysiological mechanisms of the increased incidence of cardiovascular events with glipizide.

### Strengths and Limitations

The present study had a number of strengths. Our explicit emulation of a target trial reduces the risk of biases commonly found in observational studies, including immortal time and selection biases.^42,43^ The study population was socioeconomically similar to the US population, as indicated by the Social Deprivation Index distribution. Unlike investigations that rely solely on claims data, all individuals included in this analysis had baseline measurements of glycemic control and kidney function and most had baseline measurements of low-density lipoprotein cholesterol—all important cardiovascular risk factors. Incorporation of National Death Index data allowed us to include specific causes of death as outcomes. Finally, utilization of a privacy-preserving patient linkage permitted merger of records for the same individuals across study sites, minimizing duplicate and missing data.

Findings of this study should be interpreted in the context of its limitations. It is not possible to completely exclude confounding by indication if 1 or more of the study medications were less likely to be prescribed to individuals at higher cardiovascular risk. However, the distribution of most of the known cardiovascular risk factors was similar across the comparison groups, even before any adjustments, which makes it unlikely that unmeasured risk factors that are correlated with the measured ones (eg, duration of diabetes or smoking status) were imbalanced across groups. Second, the median follow-up of approximately 3 years may be too short to detect further differences between study medications. Third, the studied drugs reflect treatment patterns in the US at the time of the study; gliclazide was not in use, and few individuals were taking glyburide. The exclusion of saxagliptin, previously found to increase the risk for HF compared with placebo,^19^ from the DPP4i group did not affect the results. Fourth, racial and ethnic diversity was limited and the majority of study individuals had private health insurance. Although study findings may not generalize to populations with different access to treatment, all sulfonylureas evaluated in the study had low-cost generics available throughout the study period. The analysis did not evaluate different doses of sulfonylureas, and it is possible that the differences in cardiovascular risk between sulfonylureas could be dose dependent.

## Conclusions

In this comparative effectiveness research study of individuals with T2D, individuals treated with glipizide as a second agent after metformin monotherapy had a higher risk of MACE-4 events than individuals treated with DPP4is. These findings suggest that glipizide may not be the optimal agent in treatment of individuals with T2D at moderate cardiovascular risk.

## Notes

Supplement 1. **eTable 1.** Specification of the Target Trial and Its Emulation in the BESTMED Database **eTable 2.** Inclusion of Variables in the MACE-4 Model **eTable 3.** Summary of Missing Baseline Covariate Data **eTable 4.** Baseline Characteristics of and Study Medication Prescribing for Eligible Individuals by Study Site **eTable 5.** Number of Events by Treatment Group and Type of Cardiovascular Outcome **eTable 6.** Sensitivity Analysis Excluding Saxagliptin **eFigure 1.** Unadjusted Survival Curves for MACE-4 by Treatment Group **eFigure 2.** Subgroup Analyses for the Primary Outcome: Risk Differences **eTable 7.** Estimated 5-Year Risks of 4-Point MACE by Treatment Group: Sensitivity Analyses

Supplement 2.
Nonauthor Collaborators

Supplement 3.
Data Sharing Statement
