# Error in Byline

**DOI:** 10.1001/jamanetworkopen.2025.51975

**Published:** 2025-12-12

In the Original Investigation titled “Cardiovascular Events in Individuals Treated With Sulfonylureas or Dipeptidyl Peptidase 4 Inhibitors,”^1^ published July 24, 2025, there was an error in the byline, in which the nonauthor collaborator group (BESTMED Study Research Group) was not listed. This article has been corrected.^1^
