# Supplementary material for: Cardiovascular Events in Individuals Treated With Sulfonylureas or Dipeptidyl Peptidase 4 Inhibitors
Source: JAMA Netw Open. 2025 Jul 24;8(7):e2523067. doi: 10.1001/jamanetworkopen.2025.23067 (PMC12290728; doi:10.1001/jamanetworkopen.2025.23067)
Supplement: Supplement 1. — eTable 1. Specification of the Target Trial and Its Emulation in the BESTMED Database eTable 2. Inclusion of Variables in the MACE-4 Model eTable 3. Summary of Missing Baseline Covariate Data eTable 4. Baseline Characteristics of and Study Medication Prescribing for Eligible Individuals by Study Site eTable 5. Number of Events by Treatment Group and Type of Cardiovascular Outcome eTable 6. Sensitivity Analysis Excluding Saxagliptin eFigure 1. Unadjusted Survival Curves for MACE-4 by Treatment Group eFigure 2. Subgroup Analyses for the Primary Outcome: Risk Differences eTable 7. Estimated 5-Year Risks of 4-Point MACE by Treatment Group: Sensitivity Analyses [file jamanetwopen-e2523067-s001.pdf]

Supplemental Online Content

Turchin A, Petito LC, Hegermiller E, et al. Cardiovascular events in individuals treated with sulfonylureas or dipeptidyl peptidase 4 inhibitors. *JAMA Netw. Open*. 2025;8(7):e2523067. doi:10.1001/jamanetworkopen.2025.23067

eTable 1. Specification of the Target Trial and Its Emulation in the BESTMED Database

eTable 2. Inclusion of Variables in the MACE-4 Model

eTable 3. Summary of Missing Baseline Covariate Data

eTable 4. Baseline Characteristics of and Study Medication Prescribing for Eligible Individuals by Study Site

eTable 5. Number of Events by Treatment Group and Type of Cardiovascular Outcome

eTable 6. Sensitivity Analysis Excluding Saxagliptin

eFigure 1. Unadjusted Survival Curves for MACE-4 by Treatment Group

eFigure 2. Subgroup Analyses for the Primary Outcome: Risk Differences

eTable 7. Estimated 5-Year Risks of 4-Point MACE by Treatment Group: Sensitivity Analyses

This supplemental material has been provided by the authors to give readers additional information about their work.

© 2025 Turchin A et al. *JAMA Network Open*.

eTable 1. Specification of the Target Trial and Its Emulation in the BESTMED Database

<table>
<tr><th>Target Trial</th><th>Emulation in the BESTMED database</th></tr>
<tr><td>Inclusion Criteria</td><td></td></tr>
<tr><td>Individuals aged ≥ 30 years old meeting the following criteria between January 1, 2014 and January 1, 2023:
Diabetes mellitus (DM) type 2
HbA1c of 7.0-11.0% (had to be measured within the past year)
Monotherapy with metformin for at least 3 months with no prior non-metformin outpatient diabetes therapy
At “moderate” risk of ASCVD
No history of CAD (includes MI, revascularization), ischemic stroke, PVD, or heart failure hospitalization
Men aged 30-34 and women aged 30-44 additionally must have history* of hypertension, hyperlipidemia, retinopathy, kidney disease or neuropathy
eGFR ≥ 45 ml/min/1.73m^2 (measured within the past 3 years)
Not pregnant
No history* of institutionalization with a diagnosis of dementia, metastatic cancer, end stage lung disease, end stage liver disease, pancreatitis, medullary thyroid cancer or pyelonephritis
Engagement with the healthcare system: enrollment in a health insurance plan for at least 12 months and / or attendance of at least one outpatient encounter in the prior 12 months</td><td>History was derived from at least 12 months of EHR and / or claims.

End stage lung or liver disease were defined as ICU admission with a primary diagnosis of a non-infectious lung or liver condition within one year of study enrollment. In addition to diagnosis, retinopathy was identified by laser photocoagulation procedure or intravitreal injection of anti-vascular endothelial growth factor (anti-VEGF) medication</td></tr>
<tr><td>Treatment Strategies</td><td></td></tr>
</table>

© 2025 Turchin A et al. *JAMA Network Open*.

Initiation of one of<br>1. DPP4 (alogliptin, linagliptin, sitagliptin or saxagliptin)<br>2. Sulfonylurea (SU) – glimepiride<br>3. SU – glipizide<br>4. SU – glyburide<br>within 1 month (30 days) of study enrollment<br><br>Initiation of a combination medication that includes metformin plus one of the above is acceptable; simultaneous initiation of multiple of the above or of one of the above in combination with a non-study medication is not acceptable.	Medication information was obtained from prescription records (healthcare delivery organizations) and prescription fills (health insurance plans).
Assignment Procedures	
Individuals are randomly assigned to one treatment strategy. Participants and their clinicians are aware of treatment assignment.	Individuals are assigned to the treatment strategy compatible with the prescription or dispensation. Assignment is assumed to be as if randomized conditional on:<br>• Demographics<br>○ Age, sex, insurance<br>○ Proportion treated with brand name vs. generic anticoagulants per site<br>○ Social deprivation index (by zip code)<br>• Labs (most recent in prior 3 months)<br>○ HbA1c, eGFR, LDL cholesterol<br>• Healthcare utilization in prior year<br>○ Outpatient visits<br>○ Number of medications<br>○ Lab results<br>○ Any hospitalization<br>• History of excess weight (BMI > 35 kg/m^2, or ICD code indicating class II obesity)<br>• Heart failure<br>• MAFLD/MASH<br>• Charlson comorbidity index<br>• Treatment with<br>○ Systemic glucocorticoids<br>○ Statins<br>○ Anti-platelets
Follow-up Period	

© 2025 Turchin A et al. *JAMA Network Open*.

Follow-up begins at assignment and ends at the earliest of the first study outcome or loss to follow-up.	Same.
Outcomes	
4-point major adverse cardiac event (MACE-4): Time to the first of a) death from cardiovascular causes as reported in the National Death Index, b) non-fatal myocardial infarction, c) non-fatal stroke and d) hospitalization for heart failure<br><br>Individual components of MACE-4.	Events were ascertained from electronic health record notes, visits or diagnosis/procedure codes.
Causal Contrast	
Intention-to-treat effect (effect of assignment)	Effect of assignment
Statistical Analysis Plan	
Comparisons of 5-year risks via ratios and differences. Risks estimated via pooled logistic regression models. 95% confidence intervals estimated via nonparametric bootstrapping.	Same, with standardization by the baseline covariates (listed above).

© 2025 Turchin A et al. *JAMA Network Open*.

eTable 2. Inclusion of Variables in the MACE-4 Model

Variable	Inclusion in model	No. (%) missing at baseline
Treatment arm	Indicator	0 (0%)
Month of cohort entry	3-knot spline	0 (0%)
Glycemic control[a]	Indicator of HbA1c range (8-8.9, 9-9.9, 10.0-11.0), treatment interaction	0 (0%)
Age	3-knot spline, treatment interaction	0 (0%)
Sex	Indicator	0 (0%)
eGFR	3-knot spline, treatment interaction	0 (0%)
Site-specific percentage of expensive anticoagulants[d]	Linear	0 (0%)
No. medications	3-knot spline	0 (0%)
No. labs	3-knot spline	0 (0%)
No. visits	3-knot spline	0 (0%)
SDI[b]	Indicator of tertile	5,199 (10.8%)
LDL[b]	3-knot spline, treatment interaction	5,742 (11.9%)
Health insurance[c]	Indicator of private insurance	2,799 (5.8%)
BMI category[b]	Indicator	7,890 (16.4%)
Cumulative burden	3-knot spline, treatment interaction, age interaction	0 (0%)
Heart failure	Indicator, treatment interaction	0 (0%)
Advanced fibrosis (FIB-4 > 2.67)	Indicator	0 (0%)
Hospitalization within prior 3 months	Indicator	0 (0%)
Steroid use within prior 3 months	Indicator	0 (0%)
Statins	Indicator	0 (0%)
Antiplatelets	Indicator	0 (0%)
Charlson comorbidity index	Modified to exclude diabetes; Indicator of 5 or greater	0 (0%)

[a]If HbA1c was unavailable, the underlying HbA1c was imputed from available glucose measurements based on BESTMED consortium-wide data. To create the reference sample, measurements from individuals with both HbA1c and blood glucose available within a 3-month period were selected. We then ranked individuals from smallest to greatest HbA1c, and plotted corresponding blood glucose measurements. Ranges were then defined to map available blood glucose measurements to underlying HbA1c.
[b]Imputed as site-specific mean value.
[c]Imputed government insurance.
[d]Site-specific proportion treated with brand name versus generic anticoagulants

© 2025 Turchin A et al. *JAMA Network Open*.

eTable 3. Summary of Missing Baseline Covariate Data

Variable	N (%) missing	Method to Handle for Main Analysis	Method to Handle for Sensitivity Analysis
LDL-C	5,742 (11.9%)	Site-specific mean imputation.	Inclusion of an indicator for observation plus observed values with site-specific mean imputation.
HbA1c	3,158 (6.6%)	HbA1c category imputed based on blood glucose measurements.	HbA1c category imputed based on blood glucose measurements.
Health insurance category	2,799 (5.8%)	Government insurance imputed.	No imputation; a category for missing insurance was included.
Social Deprivation Index (decile)	5,199 (10.8%)	Site-specific mean imputation.	No imputation; a category for missing SDI was included.

© 2025 Turchin A et al. *JAMA Network Open*.

eTable 4. Baseline Characteristics of and Study Medication Prescribing for Eligible Individuals by Study Site

	Overall	Site-1	Site-2	Site-3	Site-4	Site-5	Site-6	Site-7	Site-8	Site-9	Site-10	Site-11	Site-12
N (%)	48165	1217	14721	356	3875	394	1106	3026	2288	13468	2853	693	4168
Age (median [IQR])	61 [52, 69]	59 [52, 66]	69 [63, 73]	56 [47, 65]	59 [50, 66]	55 [48, 62]	59 [51, 66]	56 [48, 65]	59 [50, 66]	57 [50, 63]	58 [50, 66]	56 [47, 65]	58 [50, 66]
Female sex (%)	22674 (47.1)	573 (47.1)	7521 (51.1)	177 (49.7)	1786 (46.1)	184 (46.7)	489 (44.2)	1551 (51.3)	983 (43.0)	6029 (44.8)	1279 (44.8)	326 (47.0)	1776 (42.6)
Race (%)													
Asian	1108 (2.3)	88 (7.2)	390 (2.6)	12 (3.4)	86 (2.2)	15 (3.8)	31 (2.8)	151 (5.0)	41 (1.8)	18 (0.1)	60 (2.1)	29 (4.2)	187 (4.5)
Black	3824 (7.9)	126 (10.4)	2028 (13.8)	37 (10.4)	652 (16.8)	34 (8.6)	128 (11.6)	418 (13.8)	8 (0.3)	121 (0.9)	27 (0.9)	11 (1.6)	234 (5.6)
White	26132 (54.3)	959 (78.8)	9803 (66.6)	294 (82.6)	2886 (74.5)	296 (75.1)	900 (81.4)	2131 (70.4)	1937 (84.7)	586 (4.4)	2513 (88.1)	245 (35.4)	3582 (85.9)
Other	2107 (4.4)	12 (1.0)	790 (5.4)	11 (3.1)	243 (6.3)	10 (2.5)	40 (3.6)	202 (6.7)	176 (7.7)	45 (0.3)	121 (4.2)	407 (58.7)	50 (1.2)
No information	14994 (31.1)	32 (2.6)	1710 (11.6)	2 (0.6)	8 (0.2)	39 (9.9)	7 (0.6)	124 (4.1)	126 (5.5)	12698 (94.3)	132 (4.6)	1 (0.1)	115 (2.8)
Ethnicity (%)													
Hispanic	2623 (5.4)	204 (16.8)	471 (3.2)	9 (2.5)	258 (6.7)	33 (8.4)	35 (3.2)	827 (27.3)	57 (2.5)	28 (0.2)	360 (12.6)	144 (20.8)	197 (4.7)
Non-Hispanic	30407 (63.1)	792 (65.1)	12538 (85.2)	347 (97.5)	3522 (90.9)	355 (90.1)	1064 (96.2)	2106 (69.6)	2091 (91.4)	728 (5.4)	2438 (85.5)	533 (76.9)	3893 (93.4)
No information	15135 (31.4)	221 (18.2)	1712 (11.6)	0 (0.0)	95 (2.5)	6 (1.5)	7 (0.6)	93 (3.1)	140 (6.1)	12712 (94.4)	55 (1.9)	16 (2.3)	78 (1.9)
Social deprivation index percentile (%)													
1-30	13821 (32.2)	428 (36.9)	3571 (24.4)	26 (11.7)	1556 (40.3)	112 (28.4)	525 (64.6)	870 (29.8)	956 (42.3)	2783 (31.0)	35 (1.2)	263 (38.0)	2696 (64.7)
31-60	12736 (29.6)	255 (22.0)	4893 (33.4)	142 (63.7)	1060 (27.4)	147 (37.3)	135 (16.6)	946 (32.5)	1163 (51.5)	2776 (31.0)	16 (0.6)	163 (23.5)	1040 (25.0)
61-100	16409 (38.2)	476 (41.1)	6196 (42.3)	55 (24.7)	1249 (32.3)	135 (34.3)	153 (18.8)	1099 (37.7)	141 (6.2)	3406 (38.0)	2802 (98.2)	267 (38.5)	430 (10.3)

© 2025 Turchin A et al. *JAMA Network Open*.

	Overall	Site-1	Site-2	Site-3	Site-4	Site-5	Site-6	Site-7	Site-8	Site-9	Site-10	Site-11	Site-12
HbA1c (median [IQR])	7.8 [7.3, 8.5]	7.9 [7.4, 8.6]	7.7 [7.3, 8.4]	7.8 [7.3, 8.7]	7.8 [7.4, 8.6]	7.9 [7.4, 8.8]	7.9 [7.4, 8.6]	7.9 [7.4, 8.7]	7.7 [7.3, 8.4]	7.8 [7.4, 8.6]	7.8 [7.4, 8.5]	7.8 [7.3, 8.7]	7.8 [7.4, 8.4]
LDL cholesterol (median [IQR])	89 [70, 112]	89 [71, 112]	85 [67, 108]	94 [72.75, 120.25]	92 [72, 115]	89 [66, 110.75]	88 [69, 110]	89 [70, 113]	88 [71, 112]	93 [73, 115]	90 [70, 115]	93 [70, 119]	88 [70, 111]
BMI (median [IQR])	34 [29.8, 39]	32.1 [28.3, 36.6]	33 [28.5, 36.9]	36 [30, 41]	33.4 [29.2, 38.7]	35 [30, 40]	34 [30, 40]	33 [29, 38]	35 [31, 41]	34 [29, 39]	34 [30, 39]	33 [29, 38]	34 [30, 39]
Charlson comorbidity index (mean (SD))	2.13 (1.77)	2.71 (2.34)	2.36 (1.82)	2.61 (2.45)	2.09 (1.80)	2.07 (1.79)	2.17 (2.08)	1.61 (1.17)	1.90 (1.55)	1.99 (1.72)	1.79 (1.39)	2.19 (2.07)	1.84 (1.56)
Hypertension (%)	38516 (80.0)	917 (75.3)	13150 (89.3)	266 (74.7)	2991 (77.2)	287 (72.8)	784 (70.9)	2114 (69.9)	1697 (74.2)	10960 (81.4)	2057 (72.1)	453 (65.4)	2840 (68.1)
Heart failure (%)	2828 (5.9)	53 (4.4)	1303 (8.9)	26 (7.3)	218 (5.6)	17 (4.3)	64 (5.8)	87 (2.9)	152 (6.6)	565 (4.2)	136 (4.8)	28 (4.0)	179 (4.3)
Atrial fibrillation (%)	1471 (3.1)	28 (2.3)	646 (4.4)	13 (3.7)	121 (3.1)	12 (3.0)	29 (2.6)	33 (1.1)	63 (2.8)	303 (2.2)	73 (2.6)	14 (2.0)	136 (3.3)
T2DM Medication													
DPP4i	13849 (28.8)	520 (42.7)	3884 (26.4)	123 (34.6)	1437 (37.1)	54 (13.7)	227 (20.5)	758 (25.0)	236 (10.3)	5404 (40.1)	540 (18.9)	167 (24.1)	499 (12.0)
Glimepiride	14282 (29.7)	112 (9.2)	4575 (31.1)	57 (16.0)	1237 (31.9)	57 (14.5)	411 (37.2)	1038 (34.3)	765 (33.4)	3289 (24.4)	1380 (48.4)	192 (27.7)	1169 (28.0)
Glipizide	18147 (37.7)	483 (39.7)	5799 (39.4)	143 (40.2)	1048 (27.0)	258 (65.5)	399 (36.1)	981 (32.4)	1263 (55.2)	4253 (31.6)	839 (29.4)	284 (41.0)	2397 (57.5)
Glyburide	1887 (3.9)	102 (8.4)	463 (3.1)	33 (9.3)	153 (3.9)	25 (6.3)	69 (6.2)	249 (8.2)	24 (1.0)	522 (3.9)	94 (3.3)	50 (7.2)	103 (2.5)

© 2025 Turchin A et al. *JAMA Network Open*.

eTable 5. Number of Events by Treatment Group and Type of Cardiovascular Outcome

Medication Class	MI	Stroke	HF Hospitalization	CV Death	Total MACE-4 Events
DPP4i	240	285	246	27	798
Glimepiride	263	377	334	40	1014
Glipizide	361	430	402	44	1237
Glyburide	35	35	37	2	109

© 2025 Turchin A et al. *JAMA Network Open*.

eTable 6. Sensitivity Analysis Excluding Saxagliptin

Outcome	Glimepiride	Glipizide	Glyburide
MACE-4	1.06 (0.97, 1.16)	1.12 (1.04, 1.22)	1.03 (0.85, 1.25)
MI	0.96 (0.82, 1.12)	1.09 (0.95, 1.27)	1.04 (0.75, 1.52)
Ischemic stroke	1.13 (0.96, 1.29)	1.13 (0.97, 1.30)	0.88 (0.58, 1.26)
HF hospitalization	1.07 (0.94, 1.23)	1.11 (0.98, 1.30)	1.13 (0.81, 1.58)
Cardiovascular death	1.42 (0.91, 2.21)	1.07 (0.69, 1.69)	1.73 (0.73, 3.20)[1]

Values are 5-year risk ratios (95% CI).
[1]Due to limited sample size, estimates are partially adjusted for age only.

© 2025 Turchin A et al. *JAMA Network Open*.

eFigure 1. Unadjusted Survival Curves for MACE-4 by Treatment Group

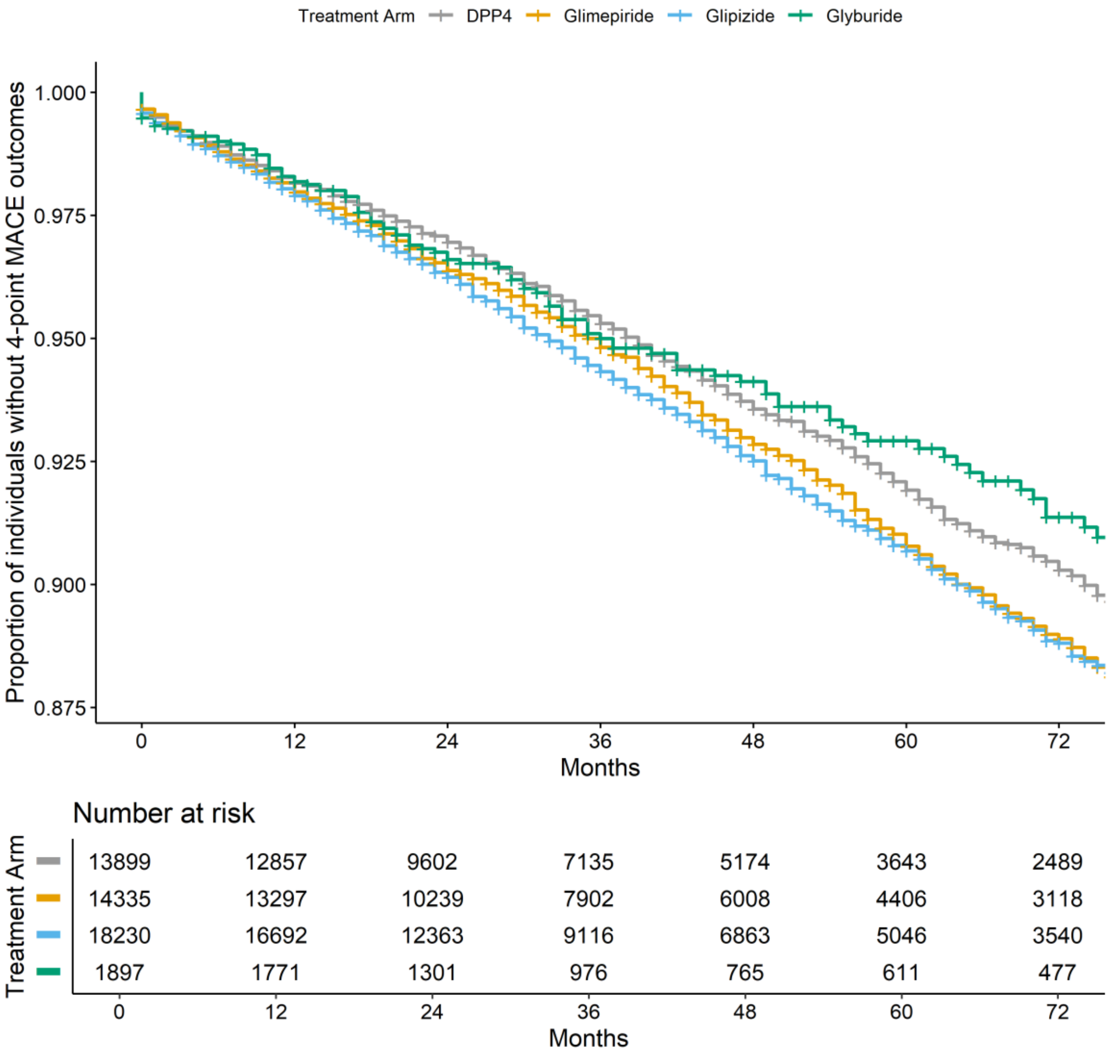

© 2025 Turchin A et al. *JAMA Network Open*.

eFigure 2. Subgroup Analyses for the Primary Outcome: Risk Differences

	Treatment Arm	n	5-year Risk (%)	Risk Difference (%)
**Overall**		**48,165**		
	DPP4	13,849	8.1 (7.5, 8.7)	0 (ref.)
	Glimepiride	14,282	8.6 (7.9, 9.2)	0.54 (-0.35, 1.25)
	Glipizide	18,147	9.1 (8.7, 9.7)	1.06 (0.23, 1.80)
	Glyburide	1,887	8.4 (6.8, 9.9)	0.29 (-1.37, 1.84)
**Sex**				
Female	DPP4	6,813	7.5 (6.7, 8.3)	0 (ref.)
	Glimepiride	6,484	8.2 (7.5, 8.9)	0.72 (-0.32, 1.62)
	Glipizide	8,444	8.7 (7.9, 9.5)	1.24 (0.29, 2.32)
	Glyburide	933	6.9 (4.8, 8.8)	-0.60 (-2.52, 1.20)
Male	DPP4	7,036	8.7 (7.8, 9.7)	0 (ref.)
	Glimepiride	7,798	9.0 (8.2, 9.8)	0.36 (-0.71, 1.56)
	Glipizide	9,703	9.5 (8.9, 10.3)	0.84 (-0.26, 1.97)
	Glyburide	954	9.6 (6.9, 11.8)	0.95 (-1.93, 3.68)
**Age**				
< 65	DPP4	9,057	5.2 (4.7, 5.9)	0 (ref.)
	Glimepiride	8,313	5.1 (4.5, 5.8)	-0.08 (-0.97, 0.70)
	Glipizide	10,838	5.4 (4.9, 6.0)	0.23 (-0.60, 0.89)
	Glyburide	1,358	5.4 (4.2, 7.0)	0.20 (-1.29, 1.88)
≥ 65	DPP4	4,792	12.7 (11.5, 14.0)	0 (ref.)
	Glimepiride	5,969	14.3 (13.3, 15.3)	1.54 (0.06, 3.04)
	Glipizide	7,309	15.1 (14.0, 16.2)	2.40 (0.82, 3.99)
	Glyburide	529	12.4 (9.0, 16.2)	-0.33 (-3.80, 3.78)
**Baseline HbA1c (%)**				
< 8	DPP4	8,075	7.9 (7.1, 8.7)	0 (ref.)
	Glimepiride	7,955	8.1 (7.2, 8.8)	0.22 (-1.02, 1.26)
	Glipizide	9,858	9.0 (8.4, 9.7)	1.16 (0.16, 2.03)
	Glyburide	950	8.4 (6.4, 10.5)	0.57 (-1.80, 2.85)
≥ 8	DPP4	5,774	8.3 (7.3, 9.3)	0 (ref.)
	Glimepiride	6,327	9.3 (8.4, 10.2)	0.98 (-0.30, 2.13)
	Glipizide	8,289	9.3 (8.6, 10.1)	1.02 (-0.07, 2.18)
	Glyburide	937	8.3 (6.2, 10.8)	0.02 (-2.29, 2.49)
**Baseline eGFR (mL/min/1.73m2)**				
< 90	DPP4	6,490	10.4 (9.4, 11.4)	0 (ref.)
	Glimepiride	7,010	10.8 (10.0, 11.5)	0.42 (-0.82, 1.50)
	Glipizide	8,942	11.8 (11.0, 12.6)	1.43 (0.23, 2.48)
	Glyburide	787	10.4 (7.9, 13.4)	0.03 (-2.66, 3.02)
≥ 90	DPP4	7,359	6.0 (5.4, 6.7)	0 (ref.)
	Glimepiride	7,272	6.6 (6.0, 7.3)	0.65 (-0.29, 1.59)
	Glipizide	9,205	6.7 (6.2, 7.4)	0.70 (-0.13, 1.55)
	Glyburide	1,100	6.7 (4.9, 8.7)	0.71 (-1.25, 2.90)

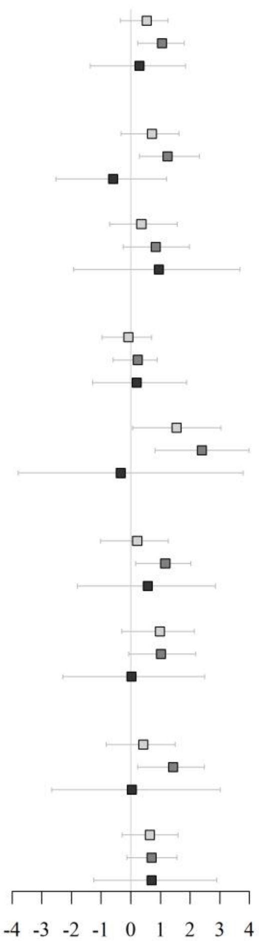

© 2025 Turchin A et al. *JAMA Network Open*.

eTable 7. Estimated 5-Year Risks of 4-Point MACE by Treatment Group: Sensitivity Analyses

**Sensitivity Analysis**	**Treatment Group**	**5-year Risk (%)**	**Risk Difference (%)**	**Risk Ratio**
Excluding Patients with History of Atrial Fibrillation or Heart Failure				
	DPP4	7.1 (6.5, 7.6)	0.00 (REF)	1.00 (REF)
	Glimepiride	7.6 (7, 8.1)	0.5 (-0.24, 1.19)	1.07 (0.97, 1.18)
	Glipizide	8.1 (7.6, 8.6)	1 (0.26, 1.73)	1.14 (1.03, 1.26)
	Glyburide	7.4 (5.9, 8.9)	0.34 (-1.37, 1.89)	1.05 (0.81, 1.28)
Censoring at competing events				
	DPP4	8.1 (7.6, 8.7)	0.00 (REF)	1.00 (REF)
	Glimepiride	8.7 (8.2, 9.4)	0.58 (-0.14, 1.27)	1.07 (0.98, 1.16)
	Glipizide	9.2 (8.7, 9.7)	1.09 (0.41, 1.77)	1.13 (1.05, 1.23)
	Glyburide	8.5 (6.7, 9.97)	0.32 (-1.3, 1.86)	1.04 (0.84, 1.24)
Adjusted for Age Only				
	DPP4	8.1 (7.5, 8.6)	0.00 (REF)	1.00 (REF)
	Glimepiride	8.6 (8.1, 9.1)	0.5 (-0.19, 1.18)	1.06 (0.98, 1.16)
	Glipizide	9.1 (8.7, 9.6)	1.04 (0.37, 1.71)	1.13 (1.04, 1.23)
	Glyburide	8.4 (6.8, 9.8)	0.31 (-1.34, 1.85)	1.04 (0.84, 1.25)
Adjusted with indicators of missingness				
	DPP4	8.2 (7.6, 8.7)	0.00 (REF)	1.00 (REF)
	Glimepiride	8.7 (8.1, 9.4)	0.50 (-0.22, 1.23)	1.06 (0.97, 1.16)
	Glipizide	9.1 (8.6, 9.6)	0.90 (0.22, 1.60)	1.11 (1.03, 1.21)
	Glyburide	8.5 (6.9, 10)	0.28 (-1.31, 1.85)	1.03 (0.84, 1.24)
Adjusted for hypertension				
	DPP4	8.1 (7.5, 8.6)	0.00 (REF)	1.00 (REF)
	Glimepiride	8.6 (8.1, 9.3)	0.5 (-0.15, 1.25)	1.07 (0.98, 1.16)
	Glipizide	9.1 (8.6, 9.7)	1.08 (0.41, 1.75)	1.13 (1.05, 1.23)
	Glyburide	8.4 (6.7, 9.9)	0.31 (-1.28, 1.87)	1.04 (0.84, 1.24)

© 2025 Turchin A et al. *JAMA Network Open*.
