# Supplementary material for: Cardiovascular Events in Individuals Treated With Sulfonylureas or Dipeptidyl Peptidase 4 Inhibitors
Source: JAMA Netw Open. 2025 Jul 24;8(7):e2523067. doi: 10.1001/jamanetworkopen.2025.23067 (PMC12290728; doi:10.1001/jamanetworkopen.2025.23067)
Supplement: Supplement 2. — Nonauthor Collaborators [file jamanetwopen-e2523067-s002.pdf]

Supplemental Online Content: Nonauthor Collaborators
*First name, last name, and suffix (if applicable) are required and will appear in PubMed.

*Group Name(s): BESTMED Study Research Group							
***First Name and Middle Initial(s)**	***Last Name**	***Suffix (eg, Jr, III)**	Academic Degrees	Institution	Location (city, state/province, country)	Role or Contribution, eg, chair, principal investigator	Group (if more than 1 Group listed in the byline) and/or Subgroup (eg, Steering Committee)
Haddy	Bah			University of Utah			BESTMED Study Research Group
Jason	Barlocker			University of Utah			BESTMED Study Research Group
Daniel L.	Bride			Intermountain Health			BESTMED Study Research Group
Elizabeth	Churchilles			University of Iowa			BESTMED Study Research Group
Megan	Douros			University of Utah			BESTMED Study Research Group
Brian	Gryzlak			University of Iowa			BESTMED Study Research Group
Channing W.	Hansen			Intermountain Health			BESTMED Study Research Group
Rachel	Hess			University of Utah			BESTMED Study Research Group
Benjamin	Horne			Intermountain Heart Institute			BESTMED Study Research Group
Jacob	Kean			University of Utah			BESTMED Study Research Group
Paul	Knudson			Medical College of Wisconsin			BESTMED Study Research Group
Victor	Melendez			Allina Health			BESTMED Study Research Group
Chris	Ortman			University of Iowa			BESTMED Study Research Group
Gi-Yung	Ryu			University of Iowa			BESTMED Study Research Group

Supplemental Online Content: Nonauthor Collaborators
*First name, last name, and suffix (if applicable) are required and will appear in PubMed.

***First Name and Middle Initial(s)**	***Last Name**	***Suffix (eg, Jr, III)**	Academic Degrees	Institution	Location (city, state/province, country)	Role or Contribution, eg, chair, principal investigator	Group (if more than 1 Group listed in the byline) and/or Subgroup (eg, Steering Committee)
Abbey	Sidebottom			Allina Health			BESTMED Study Research Group
Claire	Smith			Allina Health			BESTMED Study Research Group
Xing	Song			University of Missouri			BESTMED Study Research Group
Christine	Spinka			University of Missouri			BESTMED Study Research Group
Alexander	Stoddard			Medical College of Wisconsin			BESTMED Study Research Group
Bradley	Taylor			Medical College of Wisconsin			BESTMED Study Research Group
Jeffrey	VanWormer			Marshfield Clinic			BESTMED Study Research Group
Honglang	Zhong			Allina Health			BESTMED Study Research Group
