# Supplementary material for: Cardiovascular Events in Individuals Treated With Sulfonylureas or Dipeptidyl Peptidase 4 Inhibitors
Source: JAMA Netw Open. 2025 Jul 24;8(7):e2523067. doi: 10.1001/jamanetworkopen.2025.23067 (PMC12290728; doi:10.1001/jamanetworkopen.2025.23067)
Supplement: Supplement 3. — Data Sharing Statement [file jamanetwopen-e2523067-s003.pdf]

Data Sharing Statement

Turchin. Cardiovascular Events in Individuals Treated With Sulfonylureas or Dipeptidyl Peptidase 4 Inhibitors. *JAMA Netw Open*. Published July 24, 2025. doi:10.1001/jamanetworkopen.2025.23067

Data
Data available: No

Additional Information
Explanation for why data not available: Institutional policies of healthcare organizations that contributed data to the study do not allow us to share patient-level data.
